# The Difference in Molecular Orientation and Interphase Structure of SiO_2_/Shape Memory Polyurethane in Original, Programmed and Recovered States during Shape Memory Process

**DOI:** 10.3390/polym12091994

**Published:** 2020-09-02

**Authors:** Shuang Shi, Tao Xu, Dawei Wang, Markus Oeser

**Affiliations:** 1 College of Civil Engineering, Nanjing Forestry University, 159, Longpan Road, Nanjing 210037, China; ss@njfu.edu.cn; 2 Institute of Highway Engineering, RWTH Aachen University, Mies-van-der-Rohe-Street 1, 52074 Aachen, Germany; wang@isac.rwth-aachen.de (D.W.); oeser@isac.rwth-aachen.de (M.O.); 3 School of Transportation Science and Engineering, Harbin Institute of Technology, 73 Huanghe Road, Harbin 150090, China

**Keywords:** shape memory polyurethane, SiO_2_ particles, molecular orientation, interphase structure

## Abstract

In order to further understand the shape memory mechanism of a silicon dioxide/shape memory polyurethane (SiO_2_/SMPU) composite, the thermodynamic properties and shape memory behaviors of prepared SiO_2_/SMPU were characterized. Dynamic changes in the molecular orientation and interphase structures of SiO_2_/SMPU during a shape memory cycle were then discussed according to the small angle X-ray scattering theory, Guinier’s law, Porod approximation, and fractal dimension theorem. In this paper, a dynamic mechanical analyzer (DMA) helped to determine the glass transition start temperature (*T*_g_) by taking the onset point of the sigmoidal change in the storage modulus, while transition temperature (*T*_trans_) was defined by the peak of tan δ, then the test and the calculated results indicated that the *T*_g_ of SiO_2_/SMPU was 50.4 °C, and the *T*_trans_ of SiO_2_/SMPU was 72.18 °C. SiO_2_/SMPU showed good shape memory performance. The programmed SiO_2_/SMPU showed quite obvious microphase separation and molecular orientation. Large-size sheets and long-period structures were formed in the programmed SiO_2_/SMPU, which increases the electron density difference. Furthermore, some hard segments had been rearranged, and their gyration radii decreased. In addition, several defects formed at the interfaces of SiO_2_/SMPU, which caused the generation of space charges, thus leading to local electron density fluctuations. The blurred interphase structure and the intermediate layer formed in the programmed SiO_2_/SMPU and there was evident crystal damage and chemical bond breakage in the recovered SiO_2_/SMPU. Finally, the original and recovered SiO_2_/SMPU samples belong to the surface fractal system, but the programmed sample belongs to the mass fractal and reforms two-phase structures. This study provides an insight into the shape memory mechanism of the SiO_2_/SMPU composite.

## 1. Introduction

A shape memory polymer (SMP), as a kind of smart material, can restore its original shape due to heat, magnetism, photo, and other external stimulation [1], which includes linear or crosslinked, thermoset, or thermoplastic [2,3]. It is obvious that SMPs hold many advantages over shape memory alloy and ceramic materials such as low density, high shape recovery rate, low cost, easy molding, and so on [4]. Therefore, SMPs have attracted some quite extensive attention in recent years [5]. Shape memory polyurethane (SMPU) is one of the typical thermal-induced SMPs due to its chemical composition, which includes both soft and hard segments [6]. Weems et al. [7] modified a kind of shape memory polymer (SMP) with the renewable monomer glycerol as a more oxidatively resistant moiety. Modified polymers show an increased stability without any sacrifice to the shape memory by accelerated degradation testing. Easley et al. [8] compared the mechanical test and micro crack analysis of the SMP foam prepared by the monomer diethanolamine (DEA) and triethanolamine (TEA), and proved that DEA could effectively improve the toughness of materials and reduce micro-cracks. A thermodynamic incompatibility between the soft and hard segments leads to microphase separation and the aggregation of hard segments, which then forms a two-phase structure in SMPU [9]. The hard segment usually acts as a stationary phase in order to fix the soft segment. The soft segment, as a reversible phase, consists of a polyester or polyether polyol, which enables the SMPU to return to its original shape [10].

SMPUs have been widely studied in the past decades. It has been found that SMPUs have obvious shortcomings such as lower mechanical strength, recovery force, and poor thermal stability [11]. These factors greatly limit the application of SMPU. Recently, some studies have shown that the mechanical properties and shape recovery force of a SMPU can be increased by adding fibers, glass microspheres, silicon dioxide (SiO_2_), and so on [12,13]. Among these, the SiO_2_ particle is a typical inorganic reinforcement phase of SMPU as it demonstrates strong surface adsorption, large surface energy, high chemical purity, good dispersity, favorable thermal stability, satisfactory anti-aging performance, etc.

As a result, SiO_2_ particles used as a reinforcement material have attracted more and more attention by researchers. Through the addition of SiO_2_ particles, it is widely used in medicine, textiles, decoration, construction, and transportation, etc. [14]. Cai et al. [15] prepared a waterborne polyurethane coating by adding SiO_2_ particles and discovered that SiO_2_ particles improved the alkali and acid resistance of composites. Chen et al. [16] reported that the SiO_2_/SMPU hybrid sols exhibited uniform size distribution and good dispersion, and the applications of these hybrid sols were also investigated where it was found that the SiO_2_/SMPU sols effectively not only improved the quality of aramid fibers, but also reinforced the binding strength between the coating and the fiber. Chen et al. [17] developed a kind of wrapped material based on SiO_2_/SMPU, where its mechanical properties were improved by using the good dispersity of SiO_2_ particles in SMPU.

There are few studies about the dynamic changes in the molecular orientation of soft and hard segments of SiO_2_/SMPU. Meanwhile, there have been a few studies on the interphase structures between the SMPU and SiO_2_ particles during a shape memory cycle. Therefore, it is difficult to understand the shape memory mechanical properties of the SiO_2_/SMPU composite. The objective of this study was to more clearly understand the shape memory mechanical properties of the SiO_2_/SMPU composite, the dynamic changes in the molecular orientation, and interphase structure during a shape memory cycle, which will reveal the shape memory mechanical properties of the SMPU/SiO_2_ composite.

In this study, the SiO_2_/SMPU composite was first synthesized using an in situ polymerization method. Following this preparation, *T*_g_, *T*_trans_, and thermodynamic performance were discussed with the help of a DMA. Shape memory properties were evaluated according to the test results of the shape fixity ratio (*R*_f_) and the shape recovery ratio (*R*_r_). In the next stage, the dynamic changes in molecular orientation of hard and soft segments in the original, programmed, and recovered SiO_2_/SMPU samples were discussed, based on small angle X-ray scattering (SAXS) tests and theory. Furthermore, dynamic changes in the interphase structures between SiO_2_ particles and the SMPU as well as between soft and hard segments, were investigated according Guinier’s law, Porod approximation, and the fractal dimension theorem. This study provides an insight into the shape memory mechanism of the SiO_2_/SMPU composite.

## 2. Experimental

### 2.1. Preparation of the SiO_2_/SMPU Composite

#### 2.1.1. Raw Materials

The main materials used in this study include polyadipate-1,4-butanediol diol (PBAG, *M*_n_ = 2000, industrial grade, Asahikawa Chemical Co., Ltd., Suzhou, China), toluene diisocyanate (TDI, chemically pure, TCI Chemical Industry Development Co., Ltd., Shanghai, China), 1,4-butanediol (BDO, analytical grade, Sinopharm Chemical Reagent Co., Ltd., Shanghai, China), and SiO_2_ powder with the particle size of 16 nm (Changtai Chemical Plant, Huizhou, China).

#### 2.1.2. SiO_2_/SMPU Synthesis

SiO_2_/SMPU was prepared through uniformly dispersing 15% by volume of SiO_2_ particles in SMPU. SiO_2_ particles were dispersed in SMPU by in situ polymerization. The calculated amount of PBAG was placed in a 250 mL 4-neck round-bottom flask, which was equipped with a thermometer and mechanical stirrer as well as nitrogen inlet and outlet tubes. PBAG was dehydrated at 120 °C for 1.5 h in the vacuum environment (vacuum degree > 0.095 MPa). When the temperature was lowered to 80 °C, a calculated amount of TDI was added. The temperature was maintained at 80 °C for 2 h in nitrogen to obtain the SMPU prepolymer.

**Figure 1:**
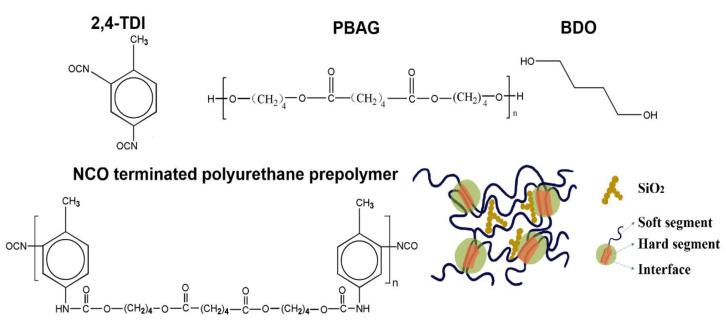
The schematic diagram of chemical structures of molecular segments in the synthesized SiO_2_/SMPU composite.

Then SiO_2_ particles were added into the prepolymer. The mixture was stirred by using a mechanical stirrer for 10 min to achieve the uniform dispersion of SiO_2_ particles. After that, when the temperature was lowered to 70 °C, the required amount of BDO as the chain extender was added dropwise into the mixture, which was rapidly stirred for 30 min. The synthesized SiO_2_/SMPU composite was immediately injected into a polytetrafluoroethylene mold, then the homogeneous mixture was cooled to room temperature and cured for 24 h without any pressure. Thus, the SiO_2_/SMPU sample was obtained after demolding. The schematic diagram of chemical structures of molecular segments in the synthesized SiO_2_/SMPU composite is shown in Figure 1.

### 2.2. Characterization Method

#### 2.2.1. DMA Test

Thermodynamic performance such as storage modulus (*E*′) and tan δ of the SiO_2_/SMPU composites were determined using a DMA (Q800, TA Instruments, New Castle, DE, USA) in the three-point bending mode. The SiO_2_/SMPU specimen size was 50 mm × 10 mm × 4.5 mm, and the specimen was tested at a loading frequency of 1 Hz, and a heating rate of 5 °C min^−1^ from 20 to 140 °C, and the amplitude was 10 μm.

#### 2.2.2. Scanning Electron Microscope (SEM) Observations

Morphology changes of pure SMPU and SiO_2_/SMPU samples in original, programmed, and recovered states were characterized using Field-Emission Scanning Electron Microscope (FESEM) (JSM-7600F, JEOL, Tokyo, Japan), respectively. The 10 mm × 10 mm × 10 mm cross-sections samples cut from the pure SMPU and SiO_2_/SMPU specimens in different states were first fixed on an aluminum stub and further sputtered with gold under vacuum conditions. Then, the sample chamber was opened to place samples. Finally, the morphologies of the samples were observed using FESEM.

#### 2.2.3. Wide Angle X-Ray Diffraction (WAXD) Test

A WAXD (Rigaku, Ultima IV, Tokyo, Japan) was used to analyze the phase microstructures of the SiO_2_/SMPU composites in the original, programmed, and recovered states, respectively. The X-Ray Diffraction (XRD) analyzer had a Cu–Ka radiation (k50.15418 nm), and the accelerating voltage and applied current were 40 kV and 30 mA, respectively. The WAXD patterns were recorded in the 2u range from 10° to 80° in the step scanning mode at a rate 2° min^−1^.

#### 2.2.4. Shape Memory Test

To endow the SMPU and SiO_2_/SMPU composite with shape memory effects, it is usually subjected to a typical shape memory cycle called programming and recovery.

To evaluate influences of SiO_2_ content on shape memory effects of SMPU, the prepared pure SMPU and SiO_2_/SMPU specimens, which were first machined into a dog bone fixed on the fixture separately, were then placed in a heating chamber without any loading. Meanwhile, the chamber was heated to *T*_trans_ and held for 20 min to make the temperature distribute uniformly in the sample.

On this basis, the sample was taken out after programming for 2 h, and cooled to room temperature. A continuous load was required during the entire process of programming and cooling, then the load was removed and the sample put back at room temperature for 24 h.

Finally, the sample stood for 24 h was put back without any loading in the chamber from the room temperature to *T*_trans_ to recovery for two hours. The pre-strain of the programmed specimen reached 25%, and the shape fixity ratio (*R*_f_) and shape recovery ratio (*R*_r_) were calculated to evaluate the shape memory effects, which were expressed as follows.

*R*_f_ = (*l*_2_ − *l*_0_)/(*l*_1_ − *l*_0_) × 100% (1)

*R*_r_ = (*l*_2_ − *l*_3_)/(*l*_2_ − *l*_0_) × 100% (2)

where *l*_0_, *l*_1_, *l*_2_, and *l*_3_ are the tagged middle lengths of the original, programmed with loading at room temperature, programmed without loading at room temperature and recovered specimens, respectively.

#### 2.2.5. SAXS Test

The changes in the molecular orientations of the original, programmed, and recovered SiO_2_/SMPU samples were characterized by using a SAXS instrument (AXS star, Bruker, Karlsruhe, Germany) with Cu-K radiation and Ni chip filtering, respectively. The accelerating voltage and applied current were 35 kV and 30 mA, respectively. The step scanning mode was used with a step interval of 0.02° at a scanning rate of 1°min^−1^. The average repeat distance of the amorphous and crystalline lamellae (semi-crystalline lamellae) of each sample was calculated by:

*d* = 2π/*q* (3)

where *d* (nm) is the lamellar repeat distance and *q* (nm^−1^) is the scalar of the scattering vector. The relationship between *q* and θ can be calculated by:

*q* = (4πsinθ)/λ (4)

where λ (nm) is the wavelength of the X-ray source.

Fractal geometry, as a natural description for disordered objects possessing dilation symmetry, seem to be self-similar after transformation of scale such as changing the magnification of a microscope. The fractal structure is normally characterized by the fractal dimension *D*, and SAXS appears to be the most appropriate technique for the determination of *D*, which is related to the scattering power law equation [18]:

*I* = *q*^−α^ (5)

where *I* is the SAXS intensity and α is the slope of the linear region of the double logarithm graph, which can be used to calculate the value of *D* of the surface(*D*_s_)/mass (*D*_m_)fractal structure.

The relation between α and *D* is followed as,

*D*_s_ = 6 − α(3 < α < 4) (6)

*D*_m_ = α(1 < α < 3) (7)

*D*_s_, which means that, if the surface is magnified, its geometric features do not change, and *D*_m_ is classified as a mass fractal and means that the density profile of the scattering objects has a self-similar nature.

## 3. Results and Discussion

### 3.1. Thermodynamic Properties

**Figure 2:**
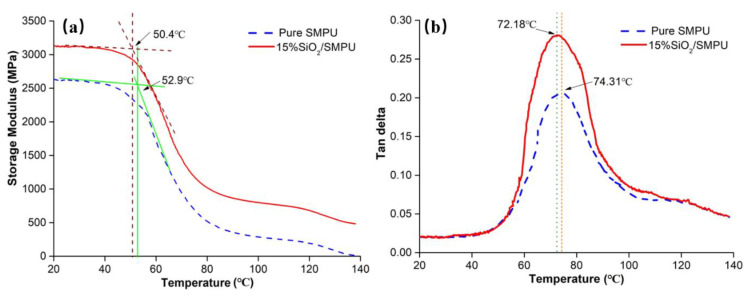
Temperature dependence of (**a**) storage modulus (E′) and (**b**) tan δ of Pure SMPU and 15% SiO_2_/SMPU.

**Figure 3:**
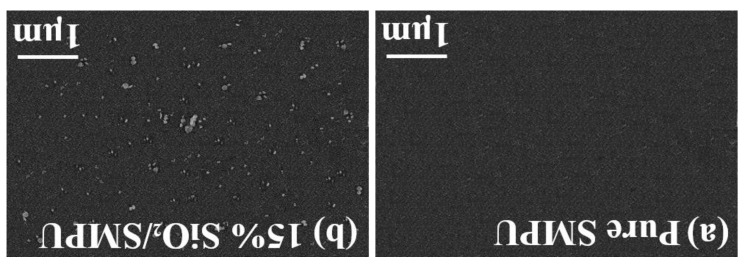
SEM images of the fractured surface of (**a**) Pure SMPU and (**b**) 15% SiO_2_/SMPU.

DMA tests were performed to discuss the thermodynamic performances and determine the *T*_g_ and *T*_trans_ of the SiO_2_/SMPU composite, as shown in Figure 2 and Figure 3 and Table 1.

We know that *T*_g_ can be determined by taking the peak of the loss modulus, the peak of tan δ or what was used in this paper, the onset point of the sigmoidal change in the storage modulus [19].

The storage modulus of the pure SMPU and SiO_2_/SMPU, which can also be called the elastic modulus, is shown in Figure 2a. Storage modulus refers to the energy stored in the process of material re-deformation due to elastic deformation, which is used to characterize the elasticity of the SiO_2_/SMPU sample. This characterization is very important for shape memory polymer materials [20]. Compared with the pure SMPU sample, the storage modulus increased with the addition of SiO_2_, which is due to the fact that when the appropriate amount of SiO_2_ particles are added, it can evenly disperse in the matrix of SMPU, form good physical entanglement to effectively improve the interface interaction with the SMPU matrix, and fill or reduce the partial voids of SMPU. It can be found that the denser the structure, the more uniform the stress, and its elastic modulus increases with the strengthening composite system.

It can be seen in Figure 2a and Table 1 that the *T*_g_ decreased slightly from 52.9 to 50.4 °C with the addition of SiO_2_, which is because the incorporated SiO_2_ disturbed the symmetry and orderness of the SMPU molecular chains, and prevented the crystallization of SMPU [11].

Using the SiO_2_/SMPU sample as an example, when the temperature range was between 20.0 and 50.4 °C, the SiO_2_/SMPU sample was in a glassy state. The storage modulus was high, with a gradual slowing downward trend, becoming more and more stable. The reason for this phenomenon is that both soft and hard segments in SiO_2_/SMPU are in a glassy crystalline state when the SiO_2_/SMPU sample is in a glassy state.

As the temperature ranged up to 50.4–87.5 °C, the SiO_2_/SMPU sample was in a transition state. The storage modulus began to decrease rapidly from 3235.29 to 489.69 MPa, and it decreased gently with the temperature rising above 87.5 °C.

It can be seen from Figure 2b that the addition of SiO_2_ enlarged the value of tan δ and the width of tan δ. On one hand, as the SiO_2_ particles have a large specific surface area, the friction force between SiO_2_ particles and molecular chain in the SMPU matrix increased, it needs more energy to make it easier to move the molecular chain segments. On the other hand, SiO_2_ dispersion was more uniform; on the basis of no agglomeration, it can effectively fill the pores of pure SMPU, which is caused by the violent reaction releasing a lot of heat during the preparation process, increasing the dense structure of SMPU, thus making the free curling of the chain segment take a longer time, so the width of the tan peak becomes larger, and the performance of the composite system is better [11].

As shown in Figure 3a,b, the microstructure of the pure SMPU and Karlsruhe SiO_2_/SMPU samples were tested. For the SiO_2_/SMPU composites, SiO_2_ agglomerates with an average size of about 70–180 nm were clearly observed. Due to the single particle size being just around 16 nm, it can be found that SiO_2_ dispersed in the SMPU matrix consisted of several primary particles, which revealed that SiO_2_ is homogenously dispersed in SMPU and further proves the results of the DMA test.

Finally, it was noted that when the temperature was 72.18 °C, the tan δ was at its largest at 0.275, which indicates that the SiO_2_/SMPU had good damping performance, meaning that when the molecular chain is subjected to external force, it will produce the largest extension. At this temperature, the material can be programmed to have a good shape memory performance [21].

Combining the results with the above discussion, when the SiO_2_/SMPU is in glass state, the molecular chains are in a static state, so the deformation is mainly due to the change in bond length and angle between atoms in the molecular chain. With the increase in temperature, it can be found that the molecular chain begins to move, and the mechanical properties of SiO_2_/SMPU begin to change when the temperature reaches the *T*_g_. Based on previous research [22], the higher the temperature, the lower the cohesive energy; when the temperature almost reached the peak of tan δ, the activity of the molecular chain was significantly enhanced, and under the action of sustained load, it was conducive to deform along the tensile direction. This indicates that the deformation by the tensile stress at the *T*_trans_ can not only extend the molecular chains, but can also achieve the best shape memory recovery performance without relaxation and loss.

In this paper, the *T*_g_ of SiO_2_/SMPU was 50.4 °C, and the temperature for programming, also considered as *T*_trans_, at which SiO_2_/SMPU had a good shape memory effect, was 72.18 °C.

### 3.2. Phase Structure Analysis

**Figure 4:**
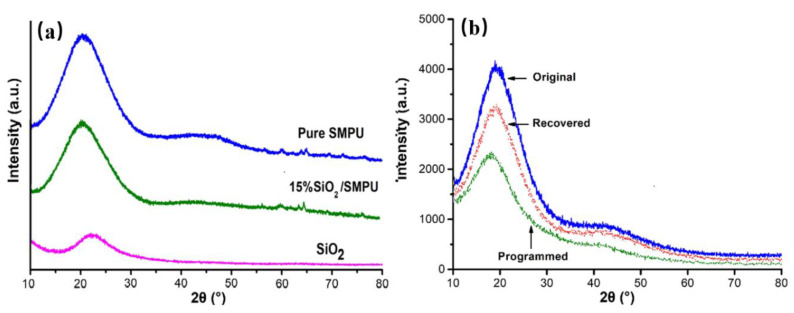
(**a**) XRD patterns of SiO_2_, Pure SMPU, and 15% SiO_2_/SMPU composites, (**b**) XRD patterns of different states of the 15% SiO_2_/SMPU composite.

To investigate the influence of SiO_2_ on the phase structures of SMPU and the changes in phase structure and crystal formation of the SiO_2_/SMPU composite in the original, programmed, and recovered states, the WAXD patterns were measured, as shown in Figure 4a,b. The grain size, full width at half maximum (FWHM), and crystallinity of pure SMPU in the original and SiO_2_/SMPU composite in three states were calculated by the Scherrer formula as given in Table 2.

As can be seen in Figure 4a, the WAXD curve of SiO_2_ showed a strong and wide diffraction peak around 2θ = 22.5°, which indicated that the SiO_2_ particles had an amorphous structure; at the same time, the Pure SMPU and SiO_2_/SMPU composite curves showed a similar broad diffraction peak around 2θ = 20.5° with higher intensities, suggesting that the pure SMPU is an amorphous polymeric material and includes a large number of amorphous phases, microcrystals, or paracrystals. As partially disordered crystals, the short and medium-range order of the lattice in microcrystals and paracrystals is present by the hydrogen bonds formed among the added SiO_2_, soft, and hard segments of SMPU, but small sizes of these kinds of crystals cannot be detected by WAXD if long-range ordering does not exist [23].

It can be seen from Figure 4a and Table 2 that the intensities of the diffraction peak of SiO_2_/SMPU at 20.5° were slightly decreased and the position of the diffraction peak was basically the same, while the crystallite size and crystallinity were also slowly reduced, and the FWHM became broader with the added SiO_2_. This can be attributed to the fact that the added SiO_2_ particles affect the motion of molecular chains in SMPU, then the orderness of chain segments is decreased and it is difficult to form a stable aggregation [11].

Figure 4b shows that the diffraction peak morphology of the programmed sample becomes weaker and wider than those of the original and recovered samples, which also slightly shifted to the lower angle. This is because that pre-stress is stored in the SiO_2_/SMPU specimen along the direction of stretching during programming; at the same time, molecular chains in soft segments are also orientated along the direction of stretching, then the regularity and orderness of soft segments in the SMPU are increased. As a result, the pre-stored residual stress in molecular chains affects the paracrystals or microcrystals in the SiO_2_/SMPU composite [24].

As shown in Table 2, the crystallinity and crystallite size of the original SiO_2_/SMPU composite were the largest, but the corresponding FWHM was the smallest; at the same time, the crystallinity and crystallite size of the programmed sample were the smallest, but the FWHM was the largest.

This is because when the temperature rises above *T*_trans_ during the programming process of the SiO_2_/SMPU composite, micro-brown movement of the molecular chain in the soft segments occurs, and the naturally curled molecular chains are oriented along the direction of stretching. The soft segments are partly melted, leading to lower crystallite size and crystalline fractures, which results in the SiO_2_/SMPU composite entering a high elastic state after being cooled to room temperature and decreasing the intensity of the diffraction peak. As a result, the programmed SiO_2_/SMPU composites have reduced crystallinity, improved mechanical properties, and restored potential molecular motion energy to restore shape [25].

When the SiO_2_/SMPU composite was again heated above *T*_trans_, the oriented molecular chains were restored to their original states due to the increase in entropy. The molecular chains shortened once more and the crystallite size and the crystallinity of the recovered SiO_2_/SMPU composite increased, leading to the diffraction peak intensity becoming stronger. However, it can be noted that the crystallinity and crystallite size of the recovered SiO_2_/SMPU composite were slightly lower than those of the original states, which indicates that the recovered SiO_2_/SMPU sample does not completely restore to its original shape. This is because the movement and agglomeration of some SiO_2_ particles lead the pore walls to be damaged, resulting in crystal loss and a decrease in the crystallinity of the recovered SiO_2_/SMPU composite [26]. Although the programming process can cause partial damage, it can improve the intermolecular interaction forces to make the SiO_2_/SMPU composites show stronger mechanical properties.

### 3.3. Shape Memory Behaviors

**Figure 5:**
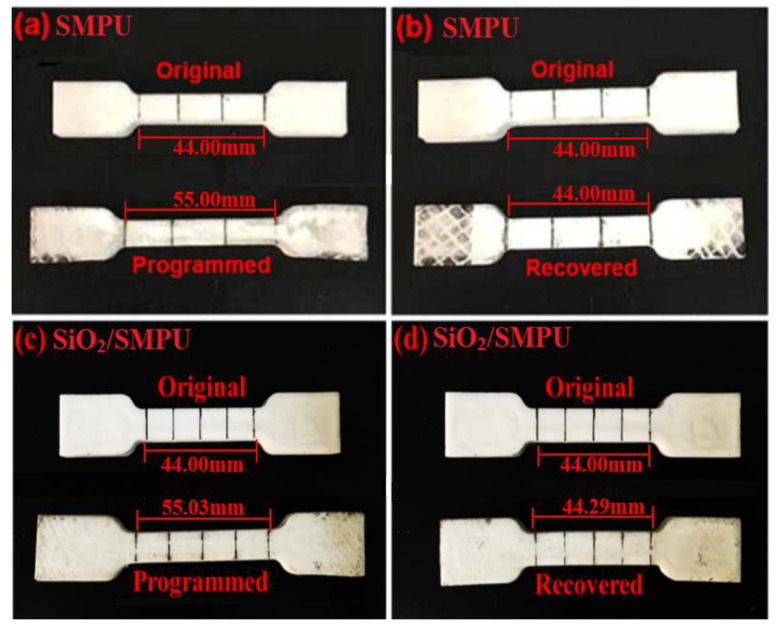
The length comparison photographs among the original, programmed, and recovered specimens of (**a**,**b**) Pure SMPU as well as (**c**,**d**) SiO_2_/SMPU composite.

**Figure 6:**
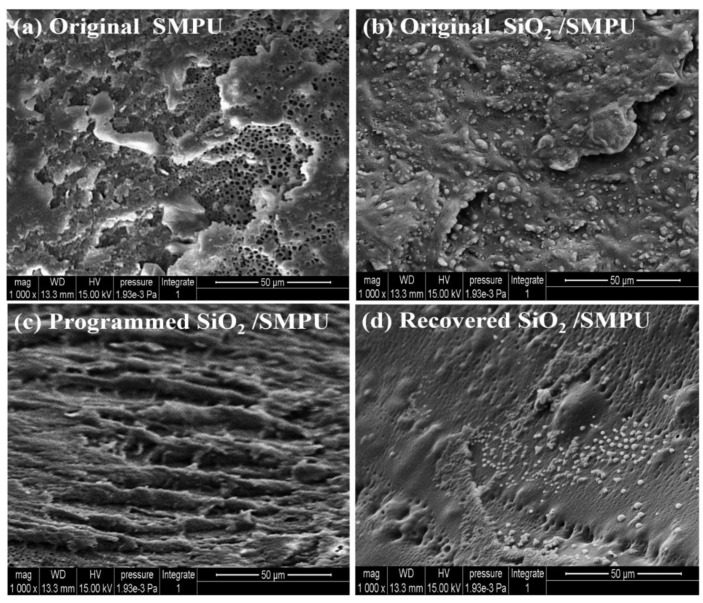
SEM images of (**a**) original SMPU, (**b**) original, (**c**) programmed, and (**d**) recovered SiO_2_/SMPU specimens during a shape memory process.

The length comparison and SEM images of the Pure SMPU and SiO_2_/SMPU specimens in the original, programmed, and recovered states are shown in Figure 5 and Figure 6. Test results of the specimen lengths, calculated *R*_f_, and *R_r_* of the SMPU and SiO_2_/SMPU specimens are summarized in Table 3.

The average values of the *R*_f_ and *R*_r_ of SiO_2_/SMPU specimens that can be found in Table 3 and Figure 5a,b were 98.16% and 97.31%, respectively, revealing that the shape memory effect of the prepared SiO_2_/SMPU was good. As shown in Figure 6a,b, the reason why the values were little less than 100% is because the SiO_2_ particles in the pores of porous SMPU affects the microphase separation of soft and hard segments. Although the movements of molecular chains were hindered and the imperfections of crystal structures in SiO_2_/SMPU were increased, it still demonstrates that the shape memory properties of the prepared SiO_2_/SMPU were good. There were also some protrusions observed on the SiO_2_/SMPU surface, because soft and hard segments aggregated to form their own microdomains, which respectively means that SiO_2_/SMPU shows crystallization properties. All of these influence the shape memory effects of the SiO_2_/SMPU composite.

The increasing length of the molecular chain as well as step-like pleats on the surface of the programmed specimen can be found in Figure 6c, which is because the molecular chain moves along the stretching direction, however, the soft and hard segments are subject to different stresses. The obvious recovered specimen surface is shown in Figure 6d, in which there were almost no step-like pleats on the surface of the recovered specimen. This indicates that the soft segment has been restored during recovery process. Although microscopic loss during the shape memory cycle leads to segment damage, the final states of the molecular chains were very close to the original curled one.

### 3.4. Molecular Orientation Characterization

**Figure 7:**
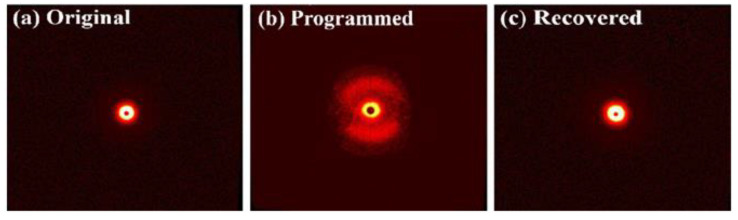
2D-SAXS patterns of (**a**) original, (**b**) programmed, and (**c**) recovered SiO_2_/SMPU samples.

To discuss the shape memory mechanism of SiO_2_/SMPU during a typical shape memory cycle, SAXS tests were performed to characterize the molecular orientation changes of SiO_2_/SMPU in the three states, respectively. Their corresponding two-dimensional (2D) SAXS patterns of the SiO_2_/SMPU samples are illustrated in Figure 7.

As demonstrated in Figure 7a,c, 2D-SAXS patterns of the original and recovered SiO_2_/SMPU had similar haloes, which indicates that the original and recovered SiO_2_/SMPU samples are isotropic [18]. Additionally, it can be seen in Figure 1 that SMPU is a typical microphase-separated polymer material, among which the hard segments—as stationary phases—are surrounded by the freely twisting soft phase. Based on previous research, this indicates that the oriented molecular chains of the soft segment in the programmed SiO_2_/SMPU are recovered to the original states and the recovered SiO_2_/SMPU sample is almost completely restored to its original state after a recovery process at 72.3 °C, which further verifies that the prepared SiO_2_/SMPU had a good shape memory performance.

In Figure 7b, the programmed SiO_2_/SMPU exhibits an anisotropic 2D-SAXS pattern following stretching to the pre-strain of 25%. The scattering intensity in the vertical direction is higher than that in the horizontal direction, which reveals that the programmed SiO_2_/SMPU shows obvious anisotropy. The reason for the anisotropy is that the amorphous molecular chains of soft segments of pure SMPU, which is seen as a kind of typical amorphous polymer, are oriented from a naturally curled state along the direction of stretching.

**Figure 8:**
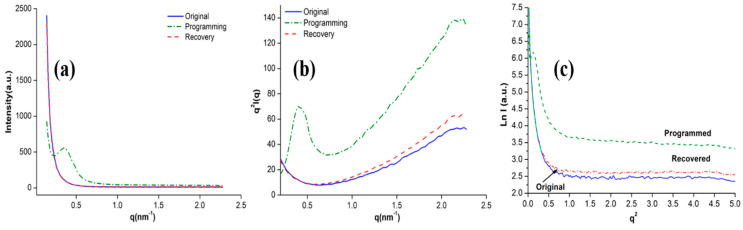
(**a**) SAXS intensity curves, (**b**) corresponding Lorentz correction curves, and (**c**) SAXS profiles of scattering curves of original, programmed, and recovered SiO_2_/SMPU samples.

In order to describe the scattering intensity changes of the SiO_2_/SMPU composite during a shape memory cycle, Figure 8 shows the SAXS intensity curves of the original, programmed, and recovered SiO_2_/SMPU samples and their corresponding Lorentz correction curves and SAXS profiles of the scattering curves.

It can be seen in Figure 8a that as the scattering vector (*q*) increased, the scattering intensity of the original and recovered SiO_2_/SMPU rapidly decreased when *q* was less than 0.5 nm^−1^, which manifested the appearance of a significant electron density difference between the crystalline and amorphous states in the SMPU matrix. This obvious electron density difference was due to the SMPU matrix being a two-phase structure polymer as well as soft and hard segments being mutually repelled and aggregated.

As the larger *q* value reflects the scattering of small-sized particles, the scattering intensity of the recovered SiO_2_/SMPU sample was slightly higher than that of the original sample when *q* was greater than 0.8 nm^−1^, which is because some SiO_2_ particles are subjected to intermolecular tensile stress during the recovery process of the SiO_2_/SMPU sample, and are moved together with molecular chains of soft segments along the direction of stretching. As a result, some pores in the SMPU are filled in, and large-size sheet structures are formed with hard segments, which slightly increases the difference in electron density [27].

Simultaneously, there an obvious characteristic peak appeared at *q* = 0.41 nm^−1^ on the scattering curve of the programmed SiO_2_/SMPU. This indicates that the molecular segments experience a change from a freely curled state to orientation along the direction of stretching during programming.

The scattering intensity of the programmed SiO_2_/SMPU was greater than that of the original and recovered SiO_2_/SMPU samples. This is because the molecular chains in the programmed sample were elongated due to stretching, with the non-periodic interval between the crystalline and amorphous states changing, and the SiO_2_ particles were filled in the SMPU pores, together with the movement of molecular chains, which increases the scattering intensity.

To be able to make peaks on the scattering intensity curves of the SiO_2_/SMPU composite more diacritical, a Lorentz correction was performed on each scattering curve, and the relationship curves of *q*^2^ *I*(*q*)-*q* were obtained as demonstrated in Figure 8b [28]. It was discovered that the characteristic scattering peak of the programmed SiO_2_/SMPU sample at *q* = 0.41 nm^−1^ was relatively high and narrow [29], which suggests that the molecular chains in the programmed SiO_2_/SMPU were stretched along the tension direction. The physically entangled SiO_2_ particles were filled in the SMPU pores again due to the movement of the molecular chains and were more uniformly dispersed in the SMPU matrix for the size of the lamellar crystals in the SMPU matrix to be relatively uniform.

According to the Bragg equation (*2d*sinθ *= n*λ), the long-period structure of the programmed SiO_2_/SMPU sample was 15.32 nm. No obvious scattering characteristic peaks were evident in the scattering intensity curves of the original and recovered SiO_2_/SMPU composites, so there were no long-period structures, which revealed that the molecular chains in the programmed SiO_2_/SMPU were oriented.

In order to further confirm the existence of molecular orientation in the programmed SiO_2_/SMPU sample, SAXS profiles of the scattering curves of the original, programmed, and recovered SiO_2_/SMPU samples according to Guinier’s law, as shown in Figure 8c. It can be noted that when *q* tended to be zero, the curve tended to be a straight line. Furthermore, the length of the straight-line part reflected the shape symmetry of the hard segment scatterer and the deviation from a spherical shape. The smaller the range of the straight-line part, the worse the particle shape symmetry [30]. The scattering curve of the programmed SiO_2_/SMPU showed a peak in the small *q* range, which indicates that hard segment scatterers in the programmed SiO_2_/SMPU sample clearly deviated from the spherical shape [31]. This deviation is due to some hard segments also being rearranged from the original spherocrystal morphology along the horizontal stretching direction during the programming of the SiO_2_/SMPU. In addition, the physical bonding of the SiO_2_ particles and hard segments may cause a decrease in shape symmetry during programming.

The calculated scatterer gyration radius (*R*_g_) of the original, programmed, and recovered SiO_2_/SMPU samples were 135, 174, and 141 nm, respectively [32]. It was noted that the scatterer R_g_ of the programmed SiO_2_/SMPU was much higher than those of the original and recovered SiO_2_/SMPU samples, and the scatterer *R*_g_ of the recovered SiO_2_/SMPU was slightly greater than that of the original sample. This subtle greater phenomenon was due to some reversible soft segments of the original SiO_2_/SMPU in the naturally curled state, surrounding and connecting hard segments and SiO_2_ particles.

Following the programming of the SiO_2_/SMPU under horizontal tensile stress, the molecular chains in soft segments were elongated, while the hard segments and SiO_2_ particles were equally dispersed in the reversible soft segments along the direction of stretching [33]. After the recovery process of SiO_2_/SMPU, some crystal structures and chemical bonds were damaged, leading to the fact that the SiO_2_/SMPU cannot completely restore to its original state, which resulted in a slight difference between the scatterer *R*_g_ of the original and recovered SiO_2_/SMPU samples.

### 3.5. Interphase Structure Changes

In order to better understand the interphase structure changes of SiO_2_/SMPU during a shape memory cycle, microstructure characteristics were discussed according to classical SAXS theory and fractal dimension theorem based on SAXS intensity curves [34].

**Figure 9:**
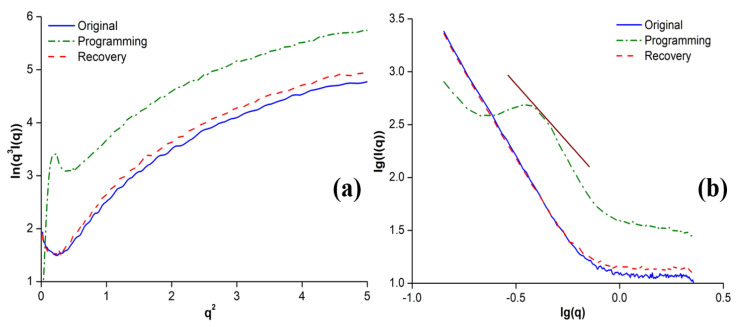
(**a**) Porod curves and (**b**) double-log SAXS patterns of original, programmed, and recovered SiO_2_/SMPU samples.

In addition, Porod approximation is often used in order to study the interphase information between different phases [35]. Porod curves and double-log SAXS patterns of the original, programmed, and recovered SiO_2_/SMPU samples are shown in Figure 9, and their fractal dimensions corresponding to double-log curves of SiO_2_/SMPU are all summarized in Table 4.

Figure 9a displays the typical Porod curves of ln[*q*^3^*I*(*q*)] − *q*^2^ based on the SAXS test results of the original, programmed, and recovered SiO_2_/SMPU samples [36]. It can be seen that the Porod curves presented some obvious positive deviations at the larger angle range, and maintained a similar change rate. Furthermore, there were electron density differences between the soft and hard segments in the SMPU matrix as well as between the SMPU matrix and SiO_2_ particles in the SiO_2_/SMPU, which led to electron density fluctuations at the above interfaces. The interphase interaction between molecular chains of the SMPU matrix and SiO_2_ particles is the main cause of a positive deviation [37].

On one hand, there are many formed defects at the interfaces between the hard and soft segments as well as between the SMPU matrix and SiO_2_ particles. The defects between them further result in the generation of space charges, which affects the electron density [38]. On the other hand, the distribution of positive and negative charges between the SiO_2_ particles and the SMPU matrix, is not a stepwise gentle transition process, but a shielding process with a “jumping” property, which causes the electrons in the interface phase to be disturbed by the charge force and localized density fluctuations occur.

Additionally, Figure 9a shows that there was a minor difference in the positive deviation between the original and recovered SiO_2_/SMPU samples. This minor difference was caused by the crystal damages and chemical bond breakages that appeared in the recovered SiO_2_/SMPU during the shape memory cycle. Furthermore, the tangled structures between the soft and hard segments in the SMPU matrix as well as between the SiO_2_ particles and SMPU matrix were changed and could not be restored to their original states, leading to differences in electron density.

A short negative deviation transition state appeared on the Porod curve of the programmed SiO_2_/SMPU sample, followed by the appearance of a positive deviation. This deviation trend was consistent with those of the original and recovered SiO_2_/SMPU samples. The occurrence of a negative deviation indicates that the blurred interface structure appeared in some states of the programmed SiO_2_/SMPU sample, and an intermediate layer appeared [24].

The appearance of this negative deviation was due to the molecular chains of the soft segments being oriented, while the hard segments and SiO_2_ particles are simultaneously moved in order to rearrange during the programming process. Additionally, some SiO_2_ particles are extruded with the pore structures of the porous SMPU being damaged. SiO_2_ particles with larger surface effects absorb some media, which reduces its surface energy, forming an interface layer on the SiO_2_ particle surface, and weakening the scattering intensity. It is demonstrated that the thermodynamic shape memory cycle will inevitably bring some microscopic damages to the SiO_2_/SMPU composite.

As shown in Figure 9b, the double-log curves of SAXS intensity of the original and recovered SiO_2_/SMPU sample presented a distinct linearity in the entire wave vector region, which showed a self-similarity. The slope values of the double-log curves were 3.71 and 3.69, respectively, and belong to the surface fractal system [30]. As the surface fractal dimension of the original SiO_2_/SMPU sample was slightly larger than that of the recovered sample, the surface of the original SiO_2_/SMPU sample was slightly rougher than that of the recovered SiO_2_/SMPU sample, as shown in Figure 3b,d. This difference between the original and recovered states was due to some SiO_2_ particles moving and filling in the SMPU matrix pores during the orientation of molecular chains in soft segments. Therefore, some physical entanglement structures were formed between the SiO_2_ particles and hard segments, which resulted in some damage to the chemical bonds and crystal structures.

In Figure 9b, the double-log curve of SAXS intensity of the programmed SiO_2_/SMPU showed the non-surface fractal phenomenon and fractal transition stage in the middle wave vector region [39], which was due to the lamellar crystal being subjected to horizontal tensile stress and being extended along the direction of stretching. At the same time, the hard segments and SiO_2_ particles were squeezed, which led to the damage of some crystal lattices and chemical bonds, resulting in forming some microcrystal domains.

When compared to the double-log curves of the original and recovered SiO_2_/SMPU samples, the entire transition state shifted with the appearance of some large particles. These particles are derived from the physical connection between the SiO_2_ particles and molecular chains in hard and soft segments. The formation of hydrogen bonds among them is one of the most important reasons why the programmed SiO_2_/SMPU is difficult to completely restore to its original shape.

In the fractal state of the programmed SiO_2_/SMPU, the slope value of the double-log curve was 2.27, which belongs to the mass fractal system. It was further confirmed that as molecular chains of reversible soft segments are stretched, and hard segments and SiO_2_ particles are also moved. As a result, the tightness degree of the SiO_2_/SMPU composite increased.

## 4. Conclusions

In this study, a SiO_2_/SMPU composite was prepared, and its *T*_t__rans_ and shape memory behaviors of SiO_2_/SMPU were characterized and the dynamic changes in molecular orientation and interphase structures during a shape memory cycle were discussed. Main conclusions were obtained as follows.

The *T*_g_ of prepared SiO_2_/SMPU was 50.4 °C, and the *T*_trans_ was 72.18 °C. The molecular chain begins to move and the mechanical properties of SiO_2_/SMPU begin to change at *T*_g_, and it has the best shape memory effect and mechanical properties at *T*_trans_.

- The XRD curves showed that the addition of SiO_2_ have little influence on Pure SMPU, but after a shape memory cycle, SiO_2_/SMPU had microscopic loss and segment damage after the recovery process, but still had a good shape memory effect, which was demonstrated in the shape memory test. After a whole cycle, the *R*_f_ and *R*_r_ of SiO_2_/SMPU could still reach above 97%.
- The molecular chains of SiO_2_/SMPU experience a process from isotropy to anisotropy, and back to isotropy during a shape memory cycle. The different scattering intensities in vertical and horizontal direction manifest that the programmed SiO_2_/SMPU has an obvious microphase separation and molecular orientation.
- The SiO_2_ particles moved with the extension of the molecular chain moving along the stretching direction, and partially filled in the pores caused by the intense reaction; some large-size sheet structures were also formed during the programming process. This further caused the change in electron density between the crystalline and amorphous regions in the SMPU matrix. According to the SAXS Lorentz correction, some long-period structure is formed in the programmed SiO_2_/SMPU, revealing the shape memory mechanism of SiO_2_/SMPU.
- The SAXS profiles of the scattering curves suggest that some hard segments in the programmed SiO_2_/SMPU were affected by the molecular chain movement to deviate from the spherical shape, and were rearranged along the stretching direction. SiO_2_ particles influence the shape symmetry of a hard segment, and the gyration radius of the hard segment scatterer was increased after the programming process of SiO_2_/SMPU.
- The Porod curves showed that some defects formed at the interfaces between hard and soft segments as well as between Pure SMPU and SiO_2_ particles. These defects produced a heterogeneous distribution of positive and negative charges at the interfaces, leading to local electron density fluctuations.
- The hard segments and SiO_2_ particles moved along the stretching direction of molecular chains of soft segments in the programming process, after that, the blurred interphase structure and intermediate layer formed in the programmed SiO_2_/SMPU. During the whole cycle of shape memory, there were some crystal damage and chemical bond breakages in the recovered SiO2/SMPU.
- Calculated fractal dimensions of original and recovered SiO_2_/SMPU samples showed a self-similarity, which is seen as a surface fractal. The programmed SiO_2_/SMPU showed a non-fractal phenomenon and fractal transition region, which is seen as mass fractal. This further confirms that hard segments and SiO_2_ particles are moved together, and reform the two-phase structure in programmed SiO_2_/SMPU.

## Figures and Tables

**Table 1:** Dynamic thermo-mechanical properties of Pure SMPU and 15% SiO_2_/SMPU.

Samples	Peak of Tan δ (°C)	E′@20 °C (MPa)	E′@120 °C (MPa)
SMPU	74.31	2631.79.	192.58
15% SiO_2_/SMPU	72.18	3235.29	489.69

**Table 2:** Phase structure parameters of different states of the SiO_2_/SMPU composite.

Phase Structure Parameters		Crystallite Size (nm)	Crystallinity	FWHM
Pure SMPU original		7.88	15.68	4.386
15% SiO_2_/SMPU	Original	7.23	14.56	4.154
Specimen	Programmed	5.21	11.03	4.849
states	Recovered	6.98	12.73	4.023

**Table 3:** Test results of the specimen lengths of SMPU and SiO_2_/SMPU in the programming and recovery process and calculated results of *R*_f_ and *R*_r_*_._*

Samples	*l*_0_ (mm)	*l*_1_ (mm)	*l*_2_ (mm)	*l*_3_ (mm)	*R*_f_ (%)	*R*_r_ (%)
SMPU	44.00	55.00	55.00	44.00	100	100
	44.00	55.02	55.02	44.00	100	100
	44.00	55.00	55.00	44.00	100	100
	44.00	55.03	54.83	44.31	98.18	97.14
SiO_2_/SMPU	44.00	54.99	54.80	44.29	98.18	97.29
	44.00	54.98	54.76	44.27	98.09	97.49

**Table 4:** Fractal dimensions corresponding to the double log curves of the original, programmed, and recovered SiO_2_/SMPU samples.

SiO_2_/SMPU Sample	α	*D*_S_	*D*_m_
Original	3.71	2.29	-
Programmed	2.27	-	2.27
Recovered	3.69	2.31	-

Note: *D*_S_ and *D*_m_ is the dimension of the surface and mass fractal structure.
